# Human endogenous retroviral elements promote genome instability via non-allelic homologous recombination

**DOI:** 10.1186/s12915-014-0074-4

**Published:** 2014-09-23

**Authors:** Ian M Campbell, Tomasz Gambin, Piotr Dittwald, Christine R Beck, Andrey Shuvarikov, Patricia Hixson, Ankita Patel, Anna Gambin, Chad A Shaw, Jill A Rosenfeld, Paweł Stankiewicz

**Affiliations:** Department of Molecular and Human Genetics, Baylor College of Medicine, One Baylor Plaza, Rm ABBR-R809, Houston, TX USA; Institute of Informatics, University of Warsaw, Warsaw, Poland; College of Inter-Faculty Individual Studies in Mathematics and Natural Sciences, University of Warsaw, Warsaw, Poland; Signature Genomic Laboratories, PerkinElmer, Inc., Spokane, WA USA; Mossakowski Medical Research Center, Polish Academy of Sciences, Warsaw, Poland

**Keywords:** HERV, copy number variation, genome instability, non-allelic homologous recombination

## Abstract

**Background:**

Recurrent rearrangements of the human genome resulting in disease or variation are mainly mediated by non-allelic homologous recombination (NAHR) between low-copy repeats. However, other genomic structures, including AT-rich palindromes and retroviruses, have also been reported to underlie recurrent structural rearrangements. Notably, recurrent deletions of Yq12 conveying azoospermia, as well as non-pathogenic reciprocal duplications, are mediated by human endogenous retroviral elements (HERVs). We hypothesized that HERV elements throughout the genome can serve as substrates for genomic instability and result in human copy-number variation (CNV).

**Results:**

We developed parameters to identify HERV elements similar to those that mediate Yq12 rearrangements as well as recurrent deletions of 3q13.2q13.31. We used these parameters to identify HERV pairs genome-wide that may cause instability. Our analysis highlighted 170 pairs, flanking 12.1% of the genome. We cross-referenced these predicted susceptibility regions with CNVs from our clinical databases for potentially HERV-mediated rearrangements and identified 78 CNVs. We subsequently molecularly confirmed recurrent deletion and duplication rearrangements at four loci in ten individuals, including reciprocal rearrangements at two loci. Breakpoint sequencing revealed clustering in regions of high sequence identity enriched in PRDM9-mediated recombination hotspot motifs.

**Conclusions:**

The presence of deletions and reciprocal duplications suggests NAHR as the causative mechanism of HERV-mediated CNV, even though the length and the sequence homology of the HERV elements are less than currently thought to be required for NAHR. We propose that in addition to HERVs, other repetitive elements, such as long interspersed elements, may also be responsible for the formation of recurrent CNVs via NAHR.

**Electronic supplementary material:**

The online version of this article (doi:10.1186/s12915-014-0074-4) contains supplementary material, which is available to authorized users.

## Background

Structural genomic rearrangements, also known as structural variants (SVs), contribute significantly to human disease and variation with locus-specific mutation rates 100- to 1,000-fold higher than for single nucleotide variation [1]. SVs include deletions, duplications, inversions and translocations, and can generally be categorized as recurrent or non-recurrent. Recurrent SVs are characterized by virtually identical size and clustered breakpoints, whereas the breakpoints of non-recurrent SVs are more variably located throughout the genome [2]. The vast majority of the described recurrent deletions and duplications occur by non-allelic homologous recombination (NAHR) between directly oriented low-copy repeats (LCRs), also known as segmental duplications.

LCRs (defined as segments at least 1 kb in length with at least 90% sequence identity and present in more than one copy) occupy approximately 5% of the human reference genome (HRG) [3]. LCRs mediating NAHR are typically 10 kb or longer and are over 95% identical to one or more other loci [4,5]. Despite the observation that most recurrent SVs are due to NAHR between LCRs, there have also been sporadic reports of recurrent SVs not flanked by LCRs. For example, recurrent translocations can be mediated by AT-rich palindromic repeats [6-8]. Another class of genomic features that may mediate NAHR events are repetitive elements, including long interspersed elements (LINEs) and human endogenous retroviruses (HERVs) [9].

Together, LINEs and HERVs constitute over 25% of the HRG [10]. Although the vast majority of these elements are fragments of the full-length element (Additional file 1: Figure S1), they present a considerably larger target for mutagenic processes. Indeed, a number of human diseases are associated with deletion alleles where both breakpoints map within LINE elements [11-13]. In contradistinction to LINE elements, relatively fewer recurrent structural rearrangements mediated by HERVs have been described. The best studied examples are recurrent deletions and duplications of Yq12.2, the former conveying complete germ-cell aplasia (also known as Sertoli cell only syndrome) [14,15]. These copy-number variations (CNVs) are caused by apparent intrachromosomal NAHR between HERV-I elements and can be identified at low levels in sperm samples from normal donors [16]. For two unrelated patients, the breakpoints of recurrent t(4;18)(q35.1;q22.3) translocations were determined by sequencing to occur within HERV-H elements [17]. Similarly, for two unrelated patients it has been suggested that the apparently same-sized 1q41q42 deletions are mediated by HERV [18]. Recently, we identified a recurrent deletion of 3q13.2q13.31 in nine unrelated patients, each with breakpoints located in HERV-H elements [19]. Taken together, these observations suggest that an as-of-yet undescribed class of recurrent structural rearrangements arise via NAHR between repetitive elements genome-wide.

HERV elements (classified by the tRNA co-opted from the host cell to prime reverse-transcription) [20] make up approximately 0.8% of the HRG and are considered the scars of viral infections and genomic integration events in the germ-line cells of our distant ancestors [10,21]. All (or the vast majority of these elements) contain sequence variants, deletions or insertions that render them incapable of transposition or infection. Despite the preponderance of inactivating mutations, phylogenetic studies suggest that HERV elements continue to undergo extensive inter-element recombination [22]. We hypothesized that analysis of the sequence characteristics and distribution of HERV elements throughout the genome would allow us to predict regions that are potentially susceptible to HERV-mediated CNVs.

## Results

### Computational prediction of HERV elements prone to recombination events

**Figure 1:**
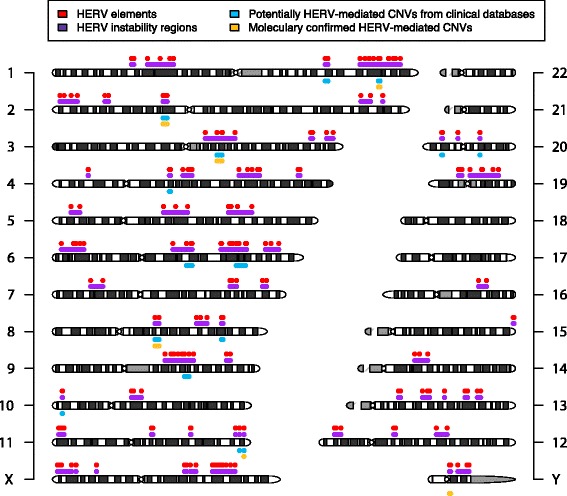
**Genome-wide map of HERV-mediated genome instability.** Chromosome ideograms with 70 predicted HERV susceptibility regions indicated in purple flanked by individual HERV elements indicated in red. Potentially HERV-mediated CNVs identified in the Baylor College of Medicine or Signature Genomics clinical databases are shown below the chromosome ideograms in cyan. HERV-mediated CNVs that have been molecularly confirmed in this study or the literature are indicated in yellow. CNV, copy-number variation; HERV, human endogenous retrovirus.

Given previous studies of NAHR events, we hypothesized that directly oriented repetitive elements with high sequence identity could mediate recurrent deletions and reciprocal duplications. To this end, we searched for highly homologous pairs of HERV elements using RepeatMasker, allowing for small gaps between annotations (see Methods). We identified 170 such HERV pairs fitting our parameters genome-wide (Additional file 2: Table S1), with the largest number on chromosome 6, with 27 pairs (Figure 1, red dots).

### Genome-wide map of HERV mediated instability

**Figure 2:**
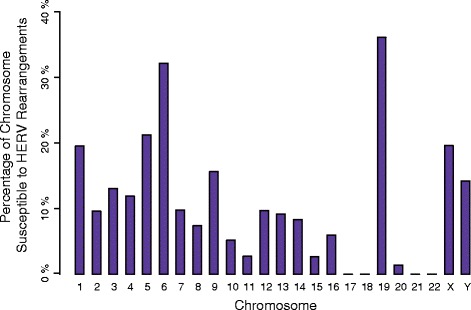
**Distribution of HERV susceptibility regions.** Percentage of each chromosome consisting of potential HERV susceptibility regions. HERV, human endogenous retrovirus.

The 170 overlapping pairs that we predicted can be condensed into 70 susceptibility regions (Figure 1, purple bars). Overall, we estimate that 375 Mb (12.1%) of the HRG could potentially undergo HERV-mediated CNV. This predicted susceptibility, genome-wide, is considerably more than the 9% predicted by Sharp *et al*. [4] and the 6% predicted by Liu *et al*. [23] for CNVs mediated by NAHR between LCRs. Chromosome 19 was determined to have the largest fraction of reference sequences within susceptibility regions as a percentage of chromosome length (Figure 2).

### Potential HERV-mediated copy-number variations

Given our list of HERV susceptibility regions, we hypothesized that interrogation of our clinical CNV databases would reveal potential HERV-mediated CNVs in patients. Thus, we cross-referenced the CNV results from 56,477 patients referred for chromosomal microarray analysis at Medical Genetics Laboratories of Baylor College of Medicine (BCM) and Signature Genomic Laboratories by oligonucleotide-based microarrays with coverage for a majority but not all of the susceptibility regions. Overall, 78 CNVs between 17 HERV pairs were seen in the combined databases (Figure 1, Additional file 3: Table S2). As expected, 1q41q42 [18], 3q13.2q13.31[19] and 8q13.3 [24] deletions were identified. The remaining 68 CNVs were flanked by 14 HERV pairs and ranged in size from 167 kb to 6.4 Mb with eight of the pairs being observed more than once. Notably, multiple deletions and the reciprocal duplications were identified at four separate loci. The most common finding was a likely benign 160-kb deletion (*n* = 15) and duplication (*n* = 27) on 10p14, involving part of a single gene for a noncoding RNA.

### Molecular analysis of predicted HERV-mediated copy-number variations

**Figure 3:**
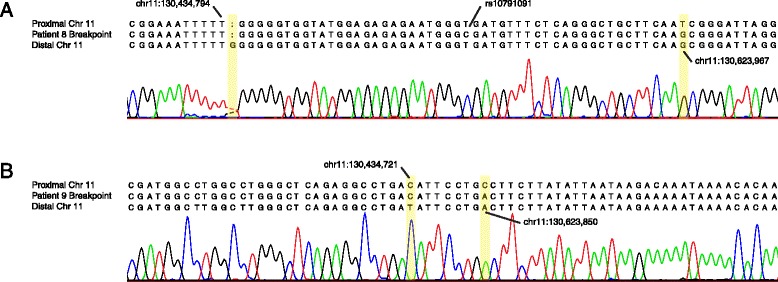
**Breakpoint analysis of HERV-mediated CNVs. (A)** Representative Sanger sequencing trace of the deletion breakpoint region of patient 8. The patient’s breakpoint sequence is presented between the proximal and distal chromosome 11 reference sequences. The informative *cis-*morphisms that define the breakpoint uncertainty region are highlighted in yellow. **(B)** An analogous presentation of a deletion breakpoint for patient 9. Chr, chromosome; CNV, copy-number variation; HERV, human endogenous retrovirus.

To test whether the CNVs identified in patients were in fact mediated by HERV elements, we mapped the CNV breakpoints by PCR and Sanger sequencing. We chose to select CNVs containing RefSeq genes to increase potential medical relevance and focused on loci seen more than once to increase our odds of true positives. We attempted to map deletions and duplications at 2p12 (77,315,373 to 78,197,976 hg19) and 11q24.3, a duplication at 2p12 (75,440,857 to 76,806,830; the reciprocal deletion was not available), as well as to map the previously suspected 1q41 deletions [18]. Overall, we tested DNA from ten individuals (including two siblings as an internal control). We designed primers based on the predicted HERV elements such that a CNV-specific junction fragment could be amplified (see Methods). In each case, we detected the predicted junction fragment, which was subsequently confirmed by Sanger sequencing (Table 1, Figure 3, Additional file 4: Figure S2). Patients tested with the same primer pairs had identically sized junction fragments, as would be expected with aligned repeats mediating an NAHR event (Additional file 4: Figure S2).

### Breakpoint analysis

Our previous analyses of nine patients with HERV-mediated deletions of 3q13.2q13.31 showed that patients with *de novo* recurrent HERV-mediated CNVs have different breakpoints [19]. Since the parents of the patients with CNVs of 1q41, 2p12 and 11q24 that we tested in this study were unavailable, breakpoint analysis gave us the opportunity to determine that these CNVs arose as independent events. Single nucleotide or indel differences (*cis*-morphisms or paralogous sequence variants) between the HERV retrotransposons enabled narrowing of the regions where the crossover event occurred to between 8 and 162 bp (Figure 3, Table 1). In each case, the patient’s breakpoint region was different, excepting two patients with 2p12 deletions who were known to be siblings.

**Table 1:** **Breakpoint coordinates and affected genes of molecularly confirmed HERV-HERV CNVs**

**Patient**	**Locus**	**Type**	**Coordinates** ^**b**^	**Size**	**RefSeq genes**	**Start maximum**	**Start minimum**	**Stop minimum**	**Stop maximum**
1	1q41	Del	chr1:222146420-223203497	1.05 Mb	*HHIPL2, TAF1A, MIA3, AIDA, BROX, FAM177B, DISP1*	222150405	222150567	223201703	223201865
2		Del				222150567	222150621	223201865	223201919
3	2p12	Dup	chr2:75440857-76806830	1.36 Mb	*FAM176A, GCFC2, MRPL19, GCFC2*	75444928	75444972	76804990	76805034
4^a^	2p12	Del	chr2:77315373-78197976	877 kb	*LRRTM4, SNAR-H*	77318677	77318740	78195410	78195473
5^a^		Del				77318677	77318740	78195410	78195473
6		Del				77318421	77318561	78195154	78195294
7		Dup				77318269	77318318	78195002	78195051
8	11q24.3	Del	chr11:130434282-130629032	189 kb	*C11orf44*	130434794	130434845	130623915	130623967
9		Del				130434721	130434729	130623842	130623850
10		Dup				130434903	130434960	130624026	130624082

^a^Patients 4 and 5 are known siblings.

^b^All coordinates are provided in the GRCh37/hg19 assembly.

Del, deletion; Dup, duplication.

### HERV elements and breakpoint distribution

**Figure 4:**
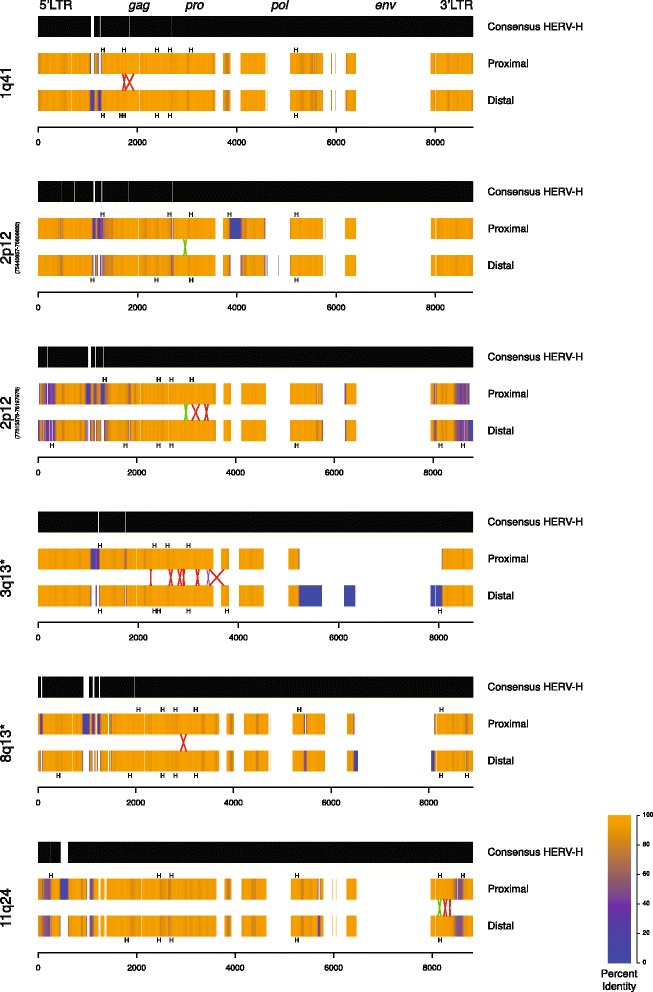
**HERV structure and breakpoint distribution of HERV-mediated CNVs.** Structures of the HERV elements involved at each locus are presented compared to the consensus HERV-H sequence from RepBase. Gaps in the consensus represent insertions in the genomic HERV elements mediating the CNVs. Gaps in the genomic HERVs represent deletions compared to the reference. The color of the genomic HERV elements denotes identity at that position when aligned with its partner element over a 50-bp window. Blue represents 0% sequence identity (i.e. caused by a large insertion or deletion) while orange represents perfect identity. The region of the crossover for each patient is presented as a colored X with the size of the X representing the uncertainty bounded by informative *cis*-morphisms. Red X’s indicate deletions; green X’s indicate duplications; the purple X represents breakpoints for two patients that occurred between the same two *cis*-morphisms. Recombination hotspot motifs in each HERV element are annotated along the HERV sequences as black H’s. The relative positions of the genes encoded in the HERV genome are annotated along the top of the first consensus. * Previously published locus. CNV, copy-number variation; HERV, human endogenous retrovirus; LTR, long tandem repeat.

We were interested in investigating the structure of the retroviral elements mediating the CNVs and the positions of the breakpoints at each locus. To this end, we multiply aligned the sequence of each HERV element with its partner and the full-length consensus HERV-H sequence from RepBase [25] (Figure 4). We repeated the same process at each locus tested in this study as well as for the autosomal loci previously reported. The HERV-H elements observed to mediate CNVs have a strikingly similar pattern of internal deletions, perhaps due to a master gene model of amplification [26]. Although 90% of HERV-H elements lack *env* sequences [27], the elements mediating CNVs additionally contain small XX and YY domain deletions, indicating a potentially close evolutionary relationship. Each element has two intact long tandem repeat (LTR) sequences flanking the internal viral sequence. Additionally, each has one or more large deletions of the *env* gene consistent with previous analysis of HERV-H sequences genome-wide [26].

### Breakpoint clustering

Visual inspection suggested that breakpoints of CNVs occurring between the same HERV elements are clustered in the same region (Figure 4). We hypothesized that this clustering is not explained by merely being confined to highly identical regions. To test this, we used a Monte Carlo approach to sample potential breakpoint positions randomly in regions of high identity (see Methods). Our analysis of the 11q24 locus revealed that breakpoints occur clustered more than by random chance (*P* = 4.4 × 10^−3^). The same analysis performed for 1q41 (*P* = 0.028), 2p12 (*P* = 0.032) and for previously determined 3q13.2q13.31 breakpoints (*P* = 2.3 × 10^−4^) identified similar clustering.

We were interested in assessing sequence features that could explain the observed clustering of breakpoints in HERV elements. Previous investigation of homologous recombination hotspots revealed a sequence motif that promotes PRDM9 (PR-Domain Containing Protein 9) binding and explains much of the increased rate of recombination [28,29]. Investigation of recurrent NAHR-mediated CNVs in patients identified a correlation between CNV frequency and the same motif [30]. We hypothesized that such recombination hotspot motifs might also be associated with the breakpoint clusters observed in the HERV elements. Assessment of the HERV element sequences revealed hotspot motifs located near the breakpoint clusters (Figure 4, black H’s). Statistical analyses of the breakpoint clusters (plus 500 bp of flanking sequence on each side) revealed that they are significantly enriched in hotspots (one-sided exact Poisson test, *P* = 7.6 × 10^−3^). The original hotspot analysis reported that a number of repetitive elements, including members of the LINE and *Alu* families, are enriched in hotspot motifs [28]. The 12 autosomal HERV-H elements observed to mediate CNVs (Figure 4) have significantly higher densities of PRDM9 hotspot motifs than the genome-wide average (Wilcox signed rank test, *P* = 1.9 × 10^−4^). The densities of hotspot motifs among the HERV-H elements observed to mediate CNVs, however, are not significantly different from the 929 other HERV-H elements with intact LTRs throughout the genome.

### HERV-mediated copy-number variations in healthy individuals

Given that we identified likely benign CNVs of 10p14 that are mediated by HERV elements in multiple individuals in our clinical cohorts, some of which were inherited from an apparently healthy parent, we hypothesized that other HERV pairs may contribute to normal genomic variation in healthy individuals. To explore this hypothesis, we designed a custom comparative genomic hybridization array (aCGH) with probes flanking the HERV elements computationally predicted to contribute to genome instability (Figure 1). We tested six healthy subjects for CNVs mediated by these HERV elements but failed to identify any predicted CNVs. Larger studies of healthy subjects will be required to investigate further the contribution of HERV-mediated CNVs to phenotypically neutral genomic variation.

## Discussion

Our combined bioinformatic prediction and molecular biology approach suggests that HERV-mediated structural rearrangements occur throughout the genome. The observation that a number of our predicted and all of our molecularly confirmed CNVs contain RefSeq genes as well as a number of Online Mendelian Inheritance in Man (OMIM) genes implies that these rearrangements have an effect on human health. Based upon the observation that breakpoints of events at the same locus in unrelated individuals occur at slightly different locations, we conclude that these CNVs arose as independent events rather than being inherited from a common ancestor. The identification of deletions and reciprocal duplications mediated by HERV elements and their association with recombination hotspot motifs further strengthens the hypothesis that these CNVs arise via NAHR. We suspect that some HERV-mediated CNVs continue to arise in the population. However, some more common HERV-mediated CNVs identified in our study (such as at 10p14, Additional file 2: Table S1) are likely to be present and segregating in the healthy population.

In addition to providing insight into a potentially new type of human recurrent structural rearrangement, our data also shed light on the substrates mediating NAHR. Cell culture experiments show that NAHR events require stretches of extreme homology or complete identity between non-allelic loci know as minimal efficient processing segments (MEPs) [31]. Estimates for MEP length in humans range from 300 to 500 bp [32]. Base mismatches and indels that interrupt the stretches of sequence identity negatively affect the efficiency of NAHR. In mice, the presence of two nucleotide mismatches results in an approximate 20-fold decrease in NAHR rate [33]. As is clear from the uncertainty regions for our patients’ breakpoints (Table 1), 300 to 500 bp of uninterrupted identity is not present. Extended analysis of the aligned sequences of the HERV elements at each breakpoint reveals a number of sequence variants and even multiple base deletions and insertions (Figure 4). Recent analysis of NAHR in humans suggests the efficiency of recombination is correlated with LCR length [5].

Although the forces described above would tend to oppose NAHR between HERV elements, we have previously identified nine events at a single locus (3q13.2q13.31) [19] and molecularly confirmed nine events throughout the genome in this study. Interestingly, the HERV-mediated events at 3q13, a previously identified translocation (t(4;18)(q35.1;q22.3)), and those described in this manuscript are all mediated by HERV-H elements. This could be largely due to the abundance of full-length HERV-H elements in the genome; HERV-H comprises approximately one-third of all *pol-*containing HERV elements in the reference sequence [17,19,27].

Dittwald and colleagues [30] identified 2,129 known pathogenic LCR-mediated CNVs from 25,144 individuals sent for chromosomal microarray analysis at a diagnostic lab (approximately 8.5%). Meanwhile, approximately 0.14% (78 of 56,477) of individuals in this study harbor HERV-mediated CNVs, although considerably different methods were used in each analysis. The rarity of HERV-mediated CNVs compared to classical LCR-mediated CNVs, despite the greater fraction of the genome potentially susceptible to HERV-mediated CNVs, could suggest that HERV elements provide less efficient MEPs. Sperm PCR analysis estimated the *de novo* mutation rate of the HERV-mediated Yq11.2 deletion to be approximately 2 × 10^−5^ per generation, as for other NAHR events [16]. Thus, the apparently lower rate of HERV-mediated CNVs genome-wide may be due to other factors. An alternative hypothesis would be that the CNVs identified for our patients are best explained by errors in DNA replication such as those proposed in the fork stalling and template switching (FoSTeS) [34] and microhomology-mediated break-induced replication (MMBIR) [35] models. Under such a hypothesis, the HERV sequences would serve as microhomology substrates to facilitate a template switch during DNA replication. However, no recurrent CNVs mediated by FoSTeS or MMBIR have been reported, nor have reciprocal deletions and duplications been described.

## Conclusions

Overall, we have shown that structural variation between HERV elements occurs throughout the genome. Given the reciprocal nature of the CNVs and association with recombination hotspots, they most likely occurred via NAHR. Because HERV elements (HERV-Hs in particular, likely due to their prevalence) provide both sequence identity and enrichment in hotspot motifs, we believe that they contribute substantially to genome instability and human disease. Although we were unable to identify HERV-mediated CNVs for six healthy subjects, HERV elements may also contribute to normal genomic variation in the population. LINE-LINE mediated CNVs have been anecdotally reported in the literature, although no systematic study of CNVs mediated by this much more abundant repetitive element are available. Nonetheless, we suspect LINEs, and indeed any repetitive element that provides the key features of homology and hotspot motifs, also promote CNV. These repeats represent an exciting area for future research.

## Methods

### Genome-wide HERV analysis

We obtained the sequences and coordinates of all HERV elements from the Fragments of Interrupted Repeats Joined by RepeatMasker ID track from the UCSC Genome Browser and selected HERV annotations not shorter than 4 kb. We then identified all pairs of HERVs located on the same chromosome, oriented in the same direction, with a distance between elements of 10 kb to 10 Mb. We reasoned that CNVs larger than 10 Mb would be highly deleterious, and this cut-off considerably decreased computation time. We aligned the HERV sequences by Smith–Waterman (local) alignment using the Biostrings package implemented in the R Statistical Programing Language with the gap Extension penalty set at −25 (other parameters were set as the defaults). We then selected HERV pairs with DNA sequence identity (calculated as the fraction of identical positions over the number of aligned positions) not less than 94% and alignment length greater than or equal to 2 kb (allowing us to exclude alignments of only LTRs). The sequence homology threshold was intended to correspond loosely to those that were utilized to generate the Segmental Duplication track on the UCSC browser and previous published analyses of LCRs that potentially mediate NAHR events [4,23,30,36]. Finally, we excluded from our analyses HERV pairs that overlapped directly oriented paralogous LCRs with identity greater than 94% and located 5 kb to 10 Mb from each other.

### Microarray analysis and database cross-reference

Oligonucleotide-based aCGH analysis was performed on DNA from patients using whole-genome microarrays custom-designed by Signature Genomics (Spokane, WA, USA) and manufactured by Agilent Technologies (Santa Clara, CA, USA) or Roche NimbleGen (Madison, WI, USA) [37,38] and designed by BCM Medical Genetics Laboratories (Houston, TX, USA) and manufactured by Agilent Technologies [39] as described previously. CNVs potentially mediated by recombination between HERV elements were identified by selecting CNVs containing one HERV each at the proximal and distal breakpoint uncertainty regions defined as the genomic intervals encompassed by the last normal and first deleted/duplicated and last deleted/duplicated and first normal probes respectively. Genomic DNA isolated from peripheral blood was obtained and subjected to further molecular testing.

### Long-range PCR and DNA sequencing

Long-range PCR primers were designed to flank the predicted HERV elements located within each patient’s breakpoint uncertainly region defined as the genomic interval encompassed by the last normal and first deleted/duplicated and last deleted/duplicated and first normal probes at the proximal and distal breakpoints, respectively. For deletions, forward and reverse primers were designed outside the proximal and distal HERV, respectively, facing in. For duplications, forward and reverse primers were designed inside the distal and proximal HERV, respectively, facing out, which would amplify tandem duplications. Primer sequences are available in Additional file 5: Table S3. Amplification of each breakpoint was performed using Takara LA *Taq* polymerase (Takara Bio, Otsu, Japan) using the manufacturer’s reaction protocol and 40 cycles with 10 minute elongation times. PCR products were treated with Illustra ExoStar (GE Healthcare Life Sciences, Piscataway, NJ, USA) to degrade deoxyribonucleotide triphosphate and primers. Sanger sequencing with the same primers used for amplification was performed on the CNV specific amplicons for each patient (Lonestar Labs, Houston, TX, USA). The breakpoint region for each patient was determined by aligning Sanger sequencing reads to the HRG obtained from the UCSC Genome Browser using the Sequencher software (Gene Codes Corporation, Ann Arbor, MI, USA). Breakpoint coordinates for each individual have been deposited in National Center for Biotechnology Information database of genomic structural variation (dbVar) and are available under the accession number [dbVar :nstd98].

### Hotspot motif analysis

We created a position weight matrix based on a previously reported recombination hotspot motif [28]. We assessed each CNV-mediating HERV element for matches to the motif’s position weight matrix and its reverse complement using the Biostrings package. We indicate the positions of strong matches (>85% of the maximum possible score) along the edge of each HERV in Figure 4. We also assessed the number of strong matches to the other 941 HERV-H elements with two intact LTR sequences throughout the genome as well as 10,000 sequences of comparable length randomly sampled from the HRG.

### Breakpoint clustering analysis

We performed Monte Carlo analysis of breakpoint clustering considering each locus with more than one breakpoint. In each case, we calculated the median distance between proximal *cis*-morphisms for all possible pairings of each observed proximal breakpoint. For example, at 3q13.2q13.31, eight different breakpoints have been observed resulting in 28 different pairs. We then randomly selected 10,000 equally sized sets of positions (i.e. 10,000 sets of eight for 3q13.2q13.31) in the same aligned HERV element where the 50-bp window of identity was greater than or equal to the minimum identity observed at the actual breakpoints (i.e. ≥92.1% at 3q13.2q13.31). We then selected the next most proximal *cis*-morphism at each position. Finally, we calculated the median distance between the selected *cis*-morphisms in each set across all sets, forming the empirical distribution.

### HERV-HERV-targeted comparative genomic hybridization for healthy subjects

Peripheral blood genomic DNA from six healthy individuals was collected in accordance with protocol H-33409 approved by the Institutional Review Board of BCM. DNA was tested using custom-designed genome-wide HERV-HERV-targeted comparative genomic hybridization 4 × 180 K microarrays (Agilent Technologies, Santa Clara, CA, USA). The genomic regions to be targeted by the array were generated automatically using scripts written in the Python programming language. Each computationally predicted directly oriented HERV pair was flanked by five oligonucleotide probes on each side to detect CNVs with both breakpoints mapping within HERV elements. Some predicted HERV elements were unable to be targeted because of genome structure or modifications to the prediction algorithm. Subsequently, for each array, one healthy individual was labeled with Cy3 and a different sex-matched healthy individual was labeled with Cy5. The labeling and hybridization procedures were performed according to the manufacturer’s protocols (Agilent Technologies, Santa Clara, CA, USA). Data were analyzed using Genomic Workbench software (Agilent Technologies, Santa Clara, CA, USA).

## Additional files

Additional file 1: Figure S1. Percentage of the reference human genome annotated as LINE or HERV elements. (A) Percentage of the genome encompassed by LINE elements as annotated in Repeatmasker, excluding elements smaller than a given size indicated on the *x*-axis. (B) Analogous analysis for HERV elements. Additional file 2: Table S1. HERV susceptibility pairs. Additional file 3: Table S2. Potentially HERV-mediated CNVs identified among patients in the BCM and Signature Genomic Laboratories clinical databases. Additional file 4: Figure S2. Representative gel electrophoresis analysis of breakpoint junctions for five individuals. Note that the sizes of the amplicons in patients 8 and 9 tested by the same primer pair are identical. kb, kilobase; Pt, patient. Additional file 5: Table S3. Sequences of breakpoint amplification primers.

## Abbreviations

aCGH: array comparative genomic hybridization
BCM: Baylor College of Medicine
bp: base pair
CNV: copy-number variation
FoSTeS: fork stalling and template switching
HERV: human endogenous retrovirus
HRG: human reference genome
kb: kilobase
LCR: low-copy repeat
LINE: long interspersed element
LTR: long tandem repeat
Mb: megabase
MEP: minimal efficient processing segment
MMBIR: microhomology-mediated break-induced replication
OMIM: Online Mendelian Inheritance in Man
NAHR: non-allelic homologous recombination
PCR: polymerase chain reaction
SV: structural variant
