# Supplementary material for: Human endogenous retroviral elements promote genome instability via non-allelic homologous recombination
Source: BMC Biol. 2014 Sep 23;12:74. doi: 10.1186/s12915-014-0074-4 (PMC4195946; doi:10.1186/s12915-014-0074-4)
Supplement: Additional file 1: Figure S1. — Percentage of the reference human genome annotated as LINE or HERV elements. (A) Percentage of the genome encompassed by LINE elements as annotated in Repeatmasker, excluding elements smaller than a given size indicated on the x-axis. (B) Analogous analysis for HERV elements. [file 12915_2014_74_MOESM1_ESM.docx]

**
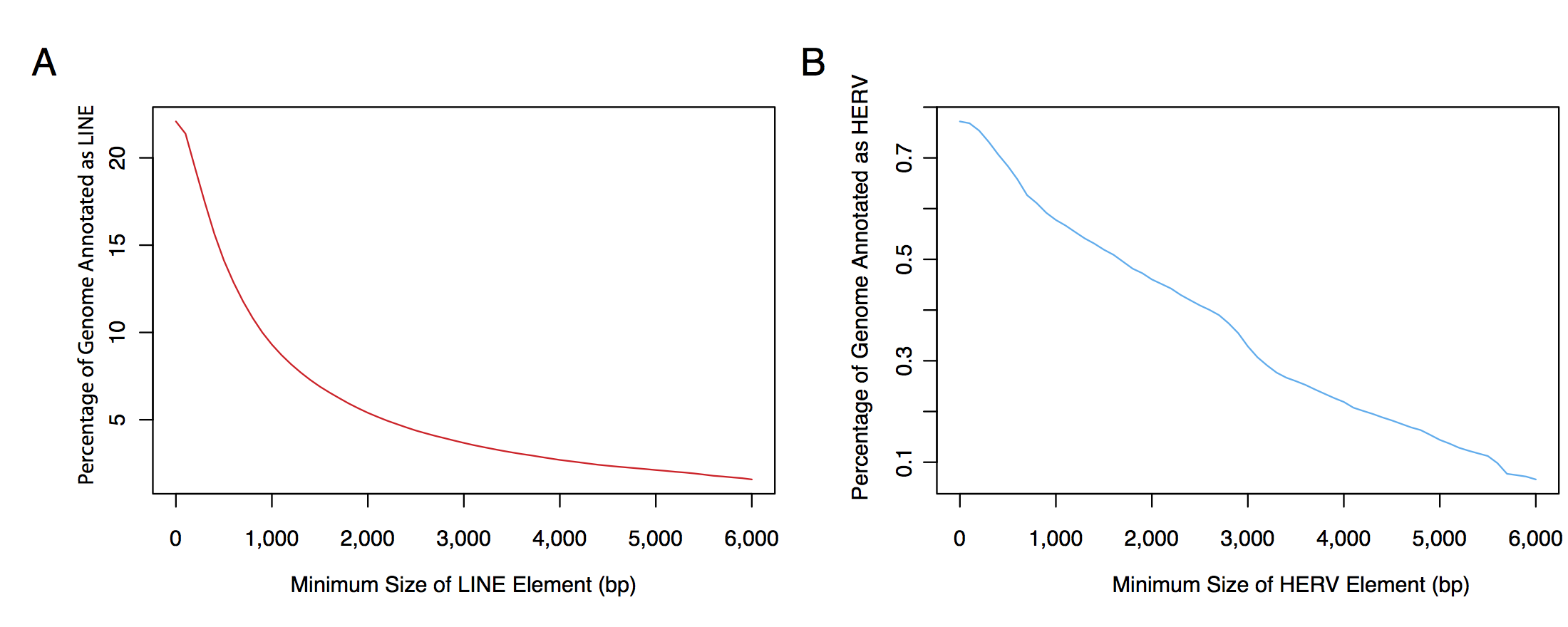
**

**Additional file 1: Figure S1**

Percentage of the Reference Human Genome Annotated as LINE or HERV elements. A. Percentage of the genome encompassed by LINE elements as annotated in Repeatmasker, excluding elements smaller than a given size indicated on the X-axis. B. Analogous analysis for HERV elements.
