# Supplementary material for: Human endogenous retroviral elements promote genome instability via non-allelic homologous recombination
Source: BMC Biol. 2014 Sep 23;12:74. doi: 10.1186/s12915-014-0074-4 (PMC4195946; doi:10.1186/s12915-014-0074-4)
Supplement: Additional file 4: Figure S2. — Representative gel electrophoresis analysis of breakpoint junctions for five individuals. Note that the sizes of the amplicons in patients 8 and 9 tested by the same primer pair are identical. kb, kilobase; Pt, patient. [file 12915_2014_74_MOESM4_ESM.doc]

**
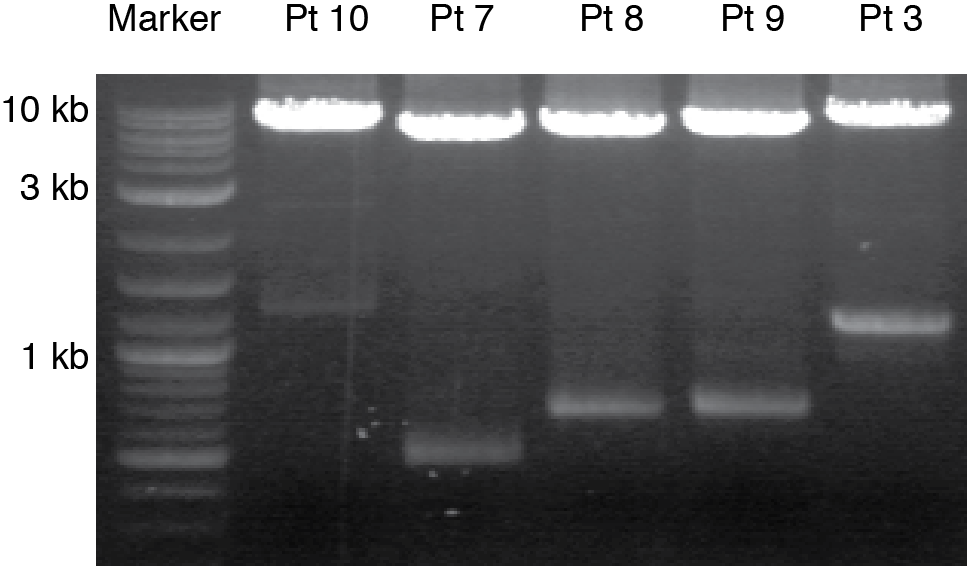
**

**Additional file 4: Figure S2**

Representative gel electrophoresis analysis of breakpoint junctions in 5 individuals. Note that the sizes of the amplicons in patients 8 and 9 tested by the same primer pair are identical.
