# Supplementary material for: Human endogenous retroviral elements promote genome instability via non-allelic homologous recombination
Source: BMC Biol. 2014 Sep 23;12:74. doi: 10.1186/s12915-014-0074-4 (PMC4195946; doi:10.1186/s12915-014-0074-4)
Supplement: Additional file 5: Table S3. — Sequences of breakpoint amplification primers. [file 12915_2014_74_MOESM5_ESM.doc]

**Additional file 5: Table S3** – Primers Sequences of Breakpoint Amplification Primers

| **Locus** | **Type** | **Coordinates** | **Size** | **Forward Primer** | **Reverse Primer** |
| --- | --- | --- | --- | --- | --- |
| 1q41 | Del | chr1:222146420-223203497 | 1.05 Mb | TACTCTTTTCGTCAACACCCTGTAGATCGTC | AGTGTTCTGCTCTCTGTGCCTCCTCTAAAA |
| 2p12 | Dup | chr2:75440857-76806830 | 1.36 Mb | GTTGCTTCTCTGATCACCCACAGATCACAT | GCTCCTGAAAGAGCTCAAAGAACACCTGAGTA |
| 2p12 | Del | chr2:77315373-78197976 | 877 kb | GGTCTTGCCTGAACAAAGAGGAGAAAAGAG | GGTTAACACACGCAATTTCTATAGGCAGTA |
|  | Dup |  |  | AAGTTGACAAGCAAGCTGACAAAGGGAAAC | GCAAACAGAAACAATCCGAAAAGAGTCAGC |
| 11q24.3 | Del | chr11:130434282-130629032 | 189 kb | GAGCATTGCCTATGAACCCCACTAGTTATG | GACAAAATCACTGAGATCTGGAAGGGAGGT |
|  | Dup |  |  | TGGAGCCTCCTCATCAAAGGTTTTTAAGTC | GTATTTCTGTCTGCTGTAGCCAAGGGAAAAG |
